# Frequency of Alloimmunization in Patients on Regular Blood Transfusion in Riyadh, Saudi Arabia: A Multicenter Retrospective Study

**DOI:** 10.3390/jcm15062340

**Published:** 2026-03-19

**Authors:** Mohammed Aldurayhim, Salman Aldosari, Muhammad Raihan Sajid, Adel Aljatham, Abdulwahab Binjomah, Ammar Alsughayir, Yazeed Alfalah, Anood Aloumi, Mubashir Hussaini, Salma Adeeb, Talah Nammor, Salah Elwishy, Imran Pukhta

**Affiliations:** 1 College of Medicine, Alfaisal University, Riyadh 11533, Saudi Arabia; m7mad-aldurihim@hotmail.com (M.A.);; 2 Department of Pathology and Laboratory Medicine, Prince Sultan Military Medical City, Riyadh 11159, Saudi Arabia; 3 King Saud Medical City, Riyadh 12746, Saudi Arabia; 4 Bacterial Diseases and Special Pathogens Department, Public Health Authority, Riyadh 13351, Saudi Arabia; 5 King Fahad Medical City, Riyadh 11316, Saudi Arabia

**Keywords:** alloimmunization, thalassemia, sickle cell anemia, blood transfusion, antibodies

## Abstract

**Background/Objectives**: Thalassemia and sickle cell anemia (SCA) patients require regular blood transfusions, a necessity that increases the risk of alloimmunization and complicates subsequent transfusion management. **Methods**: This retrospective cohort study, conducted at King Saud Medical City (KSMC) and King Fahad Medical City (KFMC) between 2018 and 2022, evaluated the frequency and risk factors of alloimmunization among 144 transfusion-dependent patients in Riyadh, Saudi Arabia. **Results**: By reviewing clinical and transfusion records alongside antibody screening results, the study found an overall alloimmunization prevalence of 20.1%. Notably, females exhibited a significantly higher rate (13.2%) compared to males (6.8%; *p* = 0.003), and younger patients (<20 years) showed a higher prevalence than older cohorts (*p* = 0.004). Analysis of ABO blood groups revealed that group A patients had a significantly lower alloimmunization rate (7.5%) compared to non-A patients (23.1%; *p* = 0.018), a finding that raises hypotheses about differential immune responsiveness but requires confirmation in larger studies. Group B showed the highest rate (35.3%), though this did not reach statistical significance after correction for multiple comparisons. RhD status was not significantly associated with alloimmunization. The most frequent alloantibodies identified were anti-E (31.3%), anti-K (12.5%), anti-D (10.4%), and anti-C (10.4%). Logistic regression further identified gender as a significant predictor (OR = 0.270; 95% CI: 0.113–0.646). **Conclusions**: Given that alloimmunization rates in Riyadh are moderately high—particularly among females and specific blood groups—and that the antibody profile (anti-E, anti-K, anti-C, anti-D) mirrors patterns seen in populations with recipient–donor ethnic mismatches, implementing extended blood group phenotyping for at least Rh (C, c, E, e) and Kell antigens prior to the first transfusion, and incorporating these findings into donor selection protocols, is critical to mitigating these risks.

## 1. Introduction

Thalassemia and sickle cell disease (SCD) are inherited hematological disorders that significantly impact red blood cell (RBC) morphology and function, posing serious public health challenges worldwide [1,2]. These conditions are especially prevalent in regions with high carrier frequencies, including Saudi Arabia and the broader Middle East [3].

Among these disorders, beta-thalassemia and SCD are of particular clinical importance due to their reliance on transfusion therapy and the associated risk of alloimmunization, which complicates disease management [4]. This study focuses on evaluating the prevalence and risk factors of alloimmunization among transfusion-dependent patients in Riyadh, Saudi Arabia. Beta-thalassemia refers to a group of autosomal recessive disorders characterized by defective synthesis of the beta-globin chains of hemoglobin, primarily due to mutations in the HBB gene [5]. This results in ineffective erythropoiesis, hemolysis, and severe anemia, necessitating lifelong transfusion therapy in cases of beta-thalassemia major [6]. In Saudi Arabia, the prevalence of beta-thalassemia ranges from 0.4% to 5.9%, underscoring its significance as a national health concern [7].

Similarly, SCD arises from a single-point mutation in the HBB gene, resulting in abnormal hemoglobin S (HbS) [8]. Under hypoxic conditions, HbS polymerizes, causing RBCs to adopt a rigid, sickle-like shape [9]. This abnormal morphology leads to hemolysis, vaso-occlusive crises, and multi-organ damage [10]. The prevalence of SCD in Saudi Arabia varies by region, with rates as high as 7% reported in certain areas, particularly the Southern Province [11].

Both disorders are associated with chronic anemia and complications that require transfusion therapy for management [12,13]. Blood transfusion is a cornerstone of treatment for beta-thalassemia major and SCD [14,15]. Regular transfusions alleviate anemia, improve oxygen-carrying capacity, and mitigate disease complications [16]. However, the risks associated with repeated transfusions, such as iron overload, transfusion reactions, and alloimmunization, present significant challenges in disease management [17].

Alloimmunization, in particular, complicates future transfusions by inducing the production of antibodies against transfused RBC antigens [18]. Alloimmunization occurs when the recipient’s immune system recognizes donor RBC antigens as foreign and produces alloantibodies [19]. Factors influencing alloimmunization include antigenic mismatches within the Rh, Kell, and other blood group systems and the recipient’s immune status [20]. Clinical consequences include delayed hemolytic transfusion reactions, refractory anemia, and difficulty in sourcing compatible blood [21].

In Saudi Arabia, studies report alloimmunization rates of 6.5–22% in beta-thalassemia patients and 11.9–19.2% in SCD patients. The most implicated antibodies include anti-E and anti-K, highlighting the need for extended antigen matching in transfusion protocols [20,22,23,24,25,26,27]. Notably, Gader et al. observed in Riyadh that none of 13 patients who received blood exclusively from Arab donors developed alloantibodies, whereas 10 of 47 patients (21.3%) who received multi-ethnic blood became alloimmunized, underscoring the critical role of donor–recipient ethnic matching in mitigating alloimmunization risk [27].

This study aims to address the knowledge gap regarding alloimmunization among transfusion-dependent patients in Riyadh, Saudi Arabia. Specifically, it investigates the prevalence of alloimmunization, identifies the most common alloantibodies encountered, and explores associations between alloimmunization and demographic and clinical variables such as gender, blood group, and age. By addressing these objectives, this study seeks to provide evidence-based insights to inform transfusion protocols, enhance patient management, and develop strategies to mitigate the risk of alloimmunization in this vulnerable population.

## 2. Methods

This study was designed as a retrospective cohort study and conducted at two tertiary medical centers in Riyadh, Saudi Arabia: King Saud Medical City (KSMC) and King Fahad Medical City (KFMC). The study period spanned from 2018 to 2022, during which clinical and transfusion records of transfusion-dependent patients were reviewed. A total of 144 patients diagnosed with thalassemia or sickle cell anemia (SCA) were included in the study. Eligibility criteria required patients to have received at least three blood transfusions within a one-year period and to have complete transfusion and clinical records available for analysis.

Data collection was performed by reviewing hematology clinic databases and blood bank records. The extracted data encompassed demographic information, including age, gender, and nationality, along with clinical variables such as diagnosis, blood group, and Rh phenotype. Transfusion history details, including the cumulative number of transfusions, age at first transfusion, and history of splenectomy, were also recorded. Laboratory results from antibody screening tests were reviewed to identify the presence and specificity of alloantibodies. Antibody screening was conducted using the indirect antiglobulin test, while the direct antiglobulin test (DAT) was applied to confirm RBC-bound antibodies when clinically indicated.

During the study period (2018–2022), both King Saud Medical City and King Fahad Medical City followed standard transfusion protocols for patients with hemoglobinopathies. RBC selection was based on ABO and RhD compatibility, with units selected to be crossmatch-compatible using standard serological techniques. Extended antigen matching for Rh (C, c, E, e) and Kell (K) antigens was not routinely performed as part of prophylactic matching protocols during this period. Instead, extended phenotyping and antibody screening were typically performed after alloantibody detection or in patients with a history of alloimmunization. For patients with identified alloantibodies, antigen-negative units were selected accordingly. The decision to perform extended phenotyping was at the discretion of the treating physician and was not standardized across all transfusion-dependent patients during the study timeframe.

The statistical analysis was conducted using SPSS software, version 31. Descriptive statistics, including frequencies, percentages, means, and medians, were used to summarize the data. Associations between alloimmunization and categorical variables, such as gender and blood group, were evaluated using chi-square tests. Continuous variables, such as age, were compared using the Wilcoxon rank-sum test due to sample size constraints. Logistic regression analysis was employed to identify independent predictors of alloimmunization, with a *p*-value of less than 0.05 considered statistically significant.

For blood group analysis, ABO (A, B, AB, O) and RhD (positive, negative) were treated as independent variables and analyzed separately, recognizing that these are genetically distinct systems encoded on different chromosomes. Percentages for categorical variables were calculated within each category (e.g., proportion of group A patients who were alloimmunized) rather than as proportions of the total study population. Chi-square tests with Yates’ correction were used for 2 × 2 comparisons, and Fisher’s exact test was applied when expected cell counts were <5. For ABO group analysis, pairwise comparisons were performed between each blood group and all others combined (e.g., A vs. non-A, B vs. non-B) to identify specific group effects. A *p*-value < 0.05 was considered statistically significant, with Bonferroni correction applied for multiple comparisons where indicated. Patients with missing data for specific variables were excluded from analyses involving those variables.

The patients’ data was collected according to ethical approval from the Institutional Review Board (IRB) at the Scientific Research Center in KSMC (approval number: H1RE-16-NOV22-02) in November 2022 and KFMC (approval number: 23-059E) in May, 2023. Because all data was obtained anonymously and did not contain any patient names or personal information, there was no need for informed consent. Patient data was extracted from files, and the blood bank database was searched for clinical information and transfusion records.

## 3. Results

The overall prevalence of alloimmunization among the 144 transfusion-dependent patients was 20.1%, with 29 patients developing alloantibodies (29/144). After verification of all data and exclusion of patients with missing values for specific analyses, the corrected results are presented below.

### 3.1. Demographic and Clinical Characteristics

Gender was significantly associated with alloimmunization. Females exhibited a higher prevalence (19/56, 33.9%) compared to males (10/88, 11.4%; *p* = 0.002). Younger patients, particularly those under 20 years of age, showed a higher prevalence of alloimmunization (15/64, 23.4%) compared to older patients (*p* = 0.004 for trend across age categories). Diagnosis (SCA vs. thalassemia) did not show a significant difference in alloimmunization rates: 16/89 (18.0%) of SCA patients were alloimmunized compared to 13/55 (23.6%) of thalassemia patients (*p* = 0.411). Nationality (Saudi vs. non-Saudi), splenectomy status, age at first transfusion, and number of transfusions per year showed no significant associations with alloimmunization (Table 1).

### 3.2. Immunohematological Results

#### 3.2.1. Antibody Screening and DAT

Antibody screening was positive in 29 patients (20.1%). Among the 53 patients who underwent direct antiglobulin testing (DAT), 13 (24.5%) tested positive, indicating the presence of autoantibodies. Among DAT-positive patients, 5/13 (38.5%) were also alloimmunized, compared to 10/40 (25.0%) of DAT-negative patients (*p* = 0.101), suggesting a possible association between autoimmunization and alloimmunization that did not reach statistical significance.

#### 3.2.2. ABO Blood Group and Alloimmunization

ABO blood group data were available for 143 patients (one patient missing). The distribution of alloimmunization by ABO group is shown in Table 2. Group A patients had the lowest alloimmunization rate (3/40, 7.5%), followed by group O (19/81, 23.5%), group AB (1/5, 20.0%), and group B (6/17, 35.3%). Pairwise comparison revealed that group A patients had a significantly lower alloimmunization rate compared to all non-A patients (3/40 [7.5%] vs. 26/103 [25.2%]; χ^2^ = 5.62, *p* = 0.018). This difference remained significant after Bonferroni correction for multiple comparisons (adjusted *p* = 0.036). Comparisons of B vs. non-B (6/17 [35.3%] vs. 20/126 [15.9%]; *p* = 0.089), O vs. non-O (19/81 [23.5%] vs. 10/62 [16.1%]; *p* = 0.30), and AB vs. non-AB (1/5 [20.0%] vs. 25/138 [18.1%]; *p* = 1.00) did not reach statistical significance.

#### 3.2.3. RhD Status and Alloimmunization

RhD status was available for 135 patients (9 not tested). RhD-positive patients (*n* = 125) had an alloimmunization rate of 20.0% (25/125), while RhD-negative patients (*n* = 10) had a rate of 40.0% (4/10). This difference was not statistically significant (*p* = 0.218), though the small number of RhD-negative patients limits interpretation.

#### 3.2.4. Number and Specificity of Alloantibodies

Among the 29 alloimmunized patients, the majority (15/29, 51.7%) developed a single alloantibody, while 10 (34.5%) developed two, 3 (10.3%) developed three, and 1 (3.4%) developed four alloantibodies. A total of 48 alloantibodies were identified. When grouped by blood group system (Table 2), antibodies against Rh system antigens were most frequent (anti-E 31.3%, anti-D 10.4%, anti-C 10.4%, anti-c 8.3%), followed by Kell system (anti-K 12.5%, anti-Kpa 6.3%), Duffy system (anti-Fya 10.4%, anti-Fyb 2.1%), Kidd system (anti-Jka 4.2%), MNS system (anti-M 2.1%), and non-specific antibodies (NSA 2.1%).

#### 3.2.5. Antibody Distribution by Diagnosis

Exploratory analysis of antibody distribution by diagnosis is presented in Table 3. Due to small sample sizes in subgroup analyses, these findings require validation in larger cohorts. Notable observations include a higher frequency of anti-D in thalassemia patients (5/26 antibodies, 19.2%) compared to SCA patients (0/29 antibodies, 0%; *p* = 0.004), and anti-c exclusively in SCA patients (4/29 antibodies, 13.8% vs. 0/26 in thalassemia, *p* = 0.111).

### 3.3. Logistic Regression Analysis

Logistic regression analysis identified gender as the only independent predictor of alloimmunization, with females at significantly higher risk (OR = 0.270; 95% CI: 0.113–0.646; *p* = 0.003). Other variables including age, age at first transfusion, cumulative transfusion units, DAT status, and splenectomy showed no significant association in the multivariate model.

Exploratory analysis of antibody distribution by diagnosis (SCA vs. thalassemia) is presented in Table 3. Due to small sample sizes in subgroup analyses, these findings should be interpreted with caution and require validation in larger cohorts. Notable observations include a higher frequency of anti-D in thalassemia patients (19.2% vs. 0% in SCA, *p* = 0.004) and anti-c exclusively in SCA patients (13.8% vs. 0%, *p* = 0.111), though the latter did not reach statistical significance.

## 4. Discussion

This retrospective study investigated the frequency of alloimmunization among 144 transfusion-dependent patients with hemoglobinopathies in Riyadh, Saudi Arabia. The overall alloimmunization prevalence of 20.1% aligns with findings from previous studies in the region, such as El-Beshlawy et al. (2020) in Egypt, who reported an 18% alloimmunization rate in β-thalassemia patients [28]. These results highlight the persistent challenge of alloimmunization in transfusion-dependent populations globally, with rates varying by region based on donor–recipient population characteristics and pre-transfusion testing protocols.

### 4.1. Gender and Alloimmunization

A significant association between female gender and higher alloimmunization risk was observed (33.9% vs. 11.4% in males, *p* = 0.002), with logistic regression confirming gender as an independent predictor (OR = 0.270; 95% CI: 0.113–0.646; *p* = 0.003). This contrasts with studies from India that found no significant gender-based differences [29,30]. The stronger association in our cohort may reflect gender-specific immune responsiveness, prior sensitization through pregnancy, or confounding by other unmeasured variables.

### 4.2. Diagnosis and Alloimmunization

No significant difference in alloimmunization rates was observed between SCA patients (18.0%) and thalassemia patients (23.6%, *p* = 0.411), consistent with some studies that suggest similar immunogenicity risks across hemoglobinopathy types when transfusion burden is comparable [20]. However, exploratory analysis of antibody specificities by diagnosis (Table 3) revealed a significantly higher frequency of anti-D in thalassemia patients (19.2% vs. 0% in SCA, *p* = 0.004) and a trend toward anti-c exclusively in SCA patients (13.8% vs. 0%, *p* = 0.111). These observations, while based on small numbers, may reflect differences in antigen exposure or immune response patterns that warrant investigation in larger cohorts.

### 4.3. ABO Blood Group and Alloimmunization

After appropriate statistical treatment of ABO and RhD as independent genetic systems, our analysis revealed that group A patients had a significantly lower alloimmunization rate (7.5%) compared to non-A patients (23.1%, *p* = 0.018). This finding raises the hypothesis that blood group A may be associated with reduced alloimmunization risk, though this observation requires validation in larger cohorts and further investigation to determine whether this represents a true protective effect, reflects underlying immune response differences, or is a chance finding in our relatively small sample. Group B showed the highest crude rate (35.3%), but this did not reach statistical significance after correction for multiple comparisons, likely due to the small number of group B patients (*n* = 17) in our cohort. RhD status alone was not significantly associated with alloimmunization, though the limited number of RhD-negative patients (*n* = 10) precludes definitive conclusions.

### 4.4. Antibody Specificities and Number

The predominance of antibodies against Rh system antigens (anti-E 31.3%, anti-D 10.4%, anti-C 10.4%, anti-c 8.3%) and Kell system antigens (anti-K 12.5%, anti-Kpa 6.3%) in this study reflects patterns reported in other Saudi studies [25,26] and internationally [31]. This profile is consistent with the immunogenicity of these antigens and the lack of routine pre-transfusion extended phenotyping during the study period. Most alloimmunized patients developed one or two antibodies (86.2%), similar to findings from Israel [31] and Japan [32].

The presence of antibodies against Duffy (Fya, Fyb), Kidd (Jka), and MNS (M) systems, while less frequent, underscores the potential value of extended phenotyping beyond Rh and Kell. Notably, the R1r (CcDee) phenotype is the most common in Saudi Arabia [33]. Recipients with this phenotype are at particular risk for anti-E alloimmunization when exposed to E-positive donor blood, highlighting the importance of E antigen matching even within ethnically similar populations.

### 4.5. DAT and Autoimmunization

Among patients who underwent DAT testing, 24.5% (13/53) tested positive for autoantibodies. The coexistence of allo- and autoantibodies in 38.5% of DAT-positive patients (compared to 25.0% of DAT-negative patients, *p* = 0.101) suggests a possible association between autoimmunization and alloimmunization that may reflect underlying immune dysregulation in multi-transfused patients [34,35]. Valsami et al. reported lower DAT positivity rates in neonatal populations, though direct comparison with our cohort is limited by different clinical contexts [29].

### 4.6. Donor Demographics and Ethnic Matching

An important contextual factor in interpreting these alloimmunization rates is the demographic composition of the blood donor pool. Gader et al. (2008) previously demonstrated in Riyadh that transfusion from ethnically matched (Arab) donors was associated with no alloimmunization (0/13 patients), whereas exposure to multi-ethnic donor blood resulted in a 21.3% alloimmunization rate [27]. However, this observation is based on only 13 patients, and larger studies are needed to confirm this finding.

Recent studies characterize the Saudi blood donor population as predominantly young male Saudi citizens [33,36]. Alalshaikh et al. (2021) reported that among blood donors in Riyadh, 98.5% were Saudi nationals [33]. While our retrospective study could not link individual recipients to specific donor demographics, the donor pool at our institutions likely reflects these published statistics.

The predominance of anti-E, anti-K, anti-C, and anti-D antibodies in our study reflects at least two contributing factors: (1) the lack of routine pre-transfusion extended phenotyping for Rh and Kell antigens, and (2) potential antigenic disparities between recipients and donors despite a predominantly Saudi donor pool. Studies from other regions have reported varying antibody patterns based on donor–recipient population genetics. Almorish et al. in Yemen highlighted anti-K as the most prevalent antibody among β-thalassemia patients [37], while Dhawan et al. (2014) in India reported different predominance patterns [38]. Wilson et al. (2023) in Egypt found no significant gender-based differences in alloimmunization, contrasting with our findings [39]. Shastry et al. emphasized the need for enhanced RhD compatibility during transfusions, while Pessoni et al. and Babiker and Elsayed reported lower anti-D prevalence rates in their populations [40,41,42]. These international comparisons underscore that absence of prophylactic matching and underlying population antigen frequency differences are distinct but additive in terms of immunogenicity. Future studies should incorporate detailed donor phenotyping and ethnicity data to better characterize the contribution of donor–recipient matching to alloimmunization risk.

#### Clinical Implications and Recommendations

Current evidence supports extended red blood cell phenotyping for patients with transfusion-dependent hemoglobinopathies. Based on international recommendations (Leal et al., 2023; Leite et al., 2024; Pirenne et al., 2021), we suggest that phenotyping should include at minimum the five most immunogenic blood group systems: Rh (C, c, D, E, e), Kell (K), Duffy (Fya, Fyb), Kidd (Jka, Jkb), and MNS (S, s) [43,44,45]. Whenever possible, this should be performed prior to the first transfusion and used to guide donor selection. Leal et al. (2023) demonstrated that prophylactic red blood cell transfusion with extended antigen matching significantly reduces alloimmunization in patients with sickle cell disease [43]. Similarly, Leite et al. (2024) emphasized the importance of RHCE and Kell genotyping to identify weak or variant antigens that may not be detected by serologic methods alone [44]. Pirenne et al. (2021) provided comprehensive guidelines for managing transfusion risk in sickle cell disease, recommending extended phenotyping as the standard of care [45]. In resource-limited settings, prioritizing Rh and Kell matching may provide the greatest immediate benefit given the predominance of antibodies against these systems. Genotyping to identify weak or variant antigens may further refine matching strategies as resources permit [43,44].

## 5. Limitations

This study has several limitations. First, the relatively small sample size (*n* = 144) limits statistical power for subgroup analyses, particularly for blood group B (*n* = 17), AB (*n* = 5), and RhD-negative (*n* = 10) patients. Second, the retrospective design precluded collection of critical variables including: (1) detailed donor phenotype and ethnicity data; (2) obstetric history in female patients to distinguish pregnancy-induced alloimmunization from transfusion-related sensitization; and (3) clinical context of transfusions (elective vs. emergency, presence of inflammatory conditions). Emerging evidence from Zheng et al. (2023) suggests that transfusions administered during proinflammatory states may carry higher alloimmunization risk, and our inability to assess this represents an important limitation [46]. Third, the high proportion of “not done” results for DAT (63.2%) and Rh phenotyping (9 patients not tested for RhD) introduces potential selection bias, though these were excluded from statistical calculations. Fourth, the lack of follow-up data prevents assessment of antibody persistence or development of delayed hemolytic transfusion reactions.

While optimizing donor–recipient matching is desirable, these strategies must be balanced against the practical realities of blood supply availability, particularly in regions with limited donor pools. The findings of this study should be interpreted with these limitations in mind.

## 6. Conclusions

This study demonstrates a 20.1% prevalence of red cell alloimmunization among transfusion-dependent patients in Riyadh, with anti-E, anti-K, anti-D, and anti-C being the most frequently identified alloantibodies. Female gender was confirmed as an independent risk factor for alloimmunization. Analysis of ABO blood groups revealed that group A patients had a significantly lower alloimmunization rate compared to non-A patients, a hypothesis-generating finding that requires validation in larger cohorts.

The predominance of Rh and Kell system antibodies reflects the absence of routine pre-transfusion extended phenotyping during the study period and underscores the potential value of prophylactic antigen matching. Based on current evidence and international recommendations, we suggest that extended red blood cell phenotyping for patients with transfusion-dependent hemoglobinopathies should include at minimum the five most immunogenic blood group systems: Rh (C, c, D, E, e), Kell (K), Duffy (Fya, Fyb), Kidd (Jka, Jkb), and MNS (S, s). Whenever possible, this should be performed prior to the first transfusion and used to guide donor selection. In resource-limited settings, prioritizing Rh and Kell matching may provide the greatest immediate benefit. Genotyping to identify weak or variant antigens may further refine matching strategies as resources permit.

Prospective studies with larger, diverse populations, detailed donor–recipient phenotyping, and longitudinal follow-up are needed to validate these findings and develop evidence-based transfusion protocols tailored to the genetic characteristics of the Saudi population while ensuring sustainable blood supply.

## Figures and Tables

**Table 1:** Demographic and Clinical Characteristics of Patients.

Category	Total N	Alloimmunized N (%) *	Non-Alloimmunized N (%) *	*p*-Value
Gender				
Male	88	10 (11.4%)	78 (88.6%)	0.002
Female	56	19 (33.9%)	37 (66.1%)	
Diagnosis				
Sickle Cell Anemia	89	16 (18.0%)	73 (82.0%)	0.411
Beta Thalassemia Major	55	13 (23.6%)	42 (76.4%)	
Nationality				
Saudi	107	19 (17.8%)	88 (82.2%)	0.226
Non-Saudi	37	10 (27.0%)	27 (73.0%)	
Splenectomy				
Yes	26	5 (19.2%)	21 (80.8%)	0.899
No	118	24 (20.3%)	94 (79.7%)	
Age Category				
<20 years	64	15 (23.4%)	49 (76.6%)	0.004 †
21–30 years	36	7 (19.4%)	29 (80.6%)	
41–50 years	41	4 (9.8%)	37 (90.2%)	
51–60 years	3	3 (100%)	0 (0%)	
Age at First Transfusion				
≤24 months	99	20 (20.2%)	79 (79.8%)	0.978
>24 months	45	9 (20.0%)	36 (80.0%)	
Number of Transfusions/Year				
3–4	1	0 (0%)	1 (100%)	0.165 ‡
5–7	12	0 (0%)	12 (100%)	
≥8	131	29 (22.1%)	102 (77.9%)	≥8

* Percentages calculated within each category; † *p*-value for trend across age categories; ‡ Fisher’s exact test.

**Table 2:** Immuno-hematological results.

Category	No.	%
Antibody screening		
Positive	29	20.10%
Negative	115	79.90%
ABO Blood Group *		
A (*n* = 40)	3	7.50%
B (*n* = 17)	6	35.30%
AB (*n* = 5)	1	20.00%
O (*n* = 81)	19	23.50%
A vs. non-A comparison		*p* = 0.018
RhD Status †		
Positive (*n* = 125)	25	20.00%
Negative (*n* = 10)	4	40.00%
		*p* = 0.218
Number of Alloantibodies per Patient		
1	15	51.70%
2	10	34.50%
3	3	10.30%
4	1	3.40%
Antibody Specificities by System (*n* = 48 antibodies)		
Rh System		
Anti-E	15	31.30%
Anti-D	5	10.40%
Anti-C	5	10.40%
Anti-c	4	8.30%
Kell System		
Anti-K	6	12.50%
Anti-Kpa	3	6.30%
Duffy System		
Anti-Fya	5	10.40%
Anti-Fyb	1	2.10%
Kidd System		
Anti-Jka	2	4.20%
MNS System		
Anti-M	1	2.10%
Non-Specific Antibody		
Anti-NSA ‡	1	2.10%
DAT Result §		
Positive	13	24.50%
Negative	40	75.50%

* ABO data available for 143 patients (1 missing). Percentages indicate proportion alloimmunized within each blood group. † RhD data available for 135 patients (9 not tested). Percentages indicate proportion alloimmunized. ‡ NSA = Non-Specific Antibody (antibody detected but specificity could not be definitively determined); § DAT analysis excludes “not done” (*n* = 91); percentages calculated among tested patients (*n* = 53).

**Table 3:** Antibody Distribution by Diagnosis (Thalassemia vs. SCA).

Antibody System/Specificity	Sickle Cell Disease (*n* = 29 Antibodies)	Thalassemia (*n* = 26 Antibodies)	Total (*n* = 55 Antibodies)	*p* Value
Rh System				
Anti-D	0 (0%)	5 (19.2%)	5 (9.1%)	0.004
Anti-C	2 (6.9%)	3 (11.5%)	5 (9.1%)	0.307
Anti-E	10 (34.5%)	5 (19.2%)	15 (27.3%)	0.632
Anti-c	4 (13.8%)	0 (0%)	4 (7.3%)	0.111
Kell System				
Anti-K	2 (6.9%)	4 (15.4%)	6 (10.9%)	0.15
Anti-Kpa	1 (3.4%)	2 (7.7%)	3 (5.5%)	0.143
Duffy System				
Anti-Fya	4 (13.8%)	1 (3.8%)	5 (9.1%)	0.394
Anti-Fyb	1 (3.4%)	0 (0%)	1 (1.8%)	0.618
Kidd System				
Anti-Jka	2 (6.9%)	0 (0%)	2 (3.6%)	0.263
MNS System				
Anti-M	0 (0%)	1 (3.8%)	1 (1.8%)	0.202
Non-Specific Antibody				
Anti-NSA *	1 (3.4%)	0 (0%)	1 (1.8%)	0.382

* NSA = Non-Specific Antibody (antibody detected but specificity could not be definitively determined).

## Data Availability

The original contributions presented in this study are included in the article. Further inquiries can be directed to the corresponding author.
